# Clinical Significance and Immunologic Landscape of a Five-IL(R)-Based Signature in Lung Adenocarcinoma

**DOI:** 10.3389/fimmu.2021.693062

**Published:** 2021-08-23

**Authors:** Tao Fan, Shize Pan, Shuo Yang, Bo Hao, Lin Zhang, Donghang Li, Qing Geng

**Affiliations:** ^1^Department of Thoracic Surgery, Renmin Hospital of Wuhan University, Wuhan, China; ^2^Department of Cardiology, Renmin Hospital of Wuhan University, Wuhan, China

**Keywords:** interleukin, lung adenocarcinoma, biomarker, immunotherapy, immunologic landscape

## Abstract

Interleukins (ILs) and interleukin receptors (ILRs) play important role in the antitumor immune response. However, the expression signature and clinical characteristics of the IL(R) family in lung adenocarcinoma (LUAD) remains unclear. The main purpose of this study was to explore the expression profile of IL(R) family genes and construct an IL(R)-based prognostic signature in LUAD. Five public datasets of 1,312 patients with LUAD were enrolled in this study. Samples from The Cancer Genome Atlas (TCGA) were used as the training set, and samples from the other four cohorts extracted from Gene Expression Omnibus (GEO) database were used as the validation set. Additionally, the profile of IL(R) family signature was explored, and the association between this signature and immunotherapy response was also analyzed. Meanwhile, the prognostic value was compared between this IL(R)-based signature and different immunotherapy markers. A signature based on five identified IL(R)s (IL7R, IL5RA, IL20RB, IL11, IL22RA1) was constructed using the TCGA dataset through univariate/multivariable Cox proportional hazards regression and least absolute shrinkage and selection operator (LASSO) Cox analysis. These cases with LUAD were stratified into high- and low-risk group according to the risk score. This signature showed a strong prognostic ability, which was verified by the five independent cohorts and clinical subtypes. The IL(R)-based models presented unique characteristics in terms of immune cell infiltration and immune inflammation profile in tumor microenvironment (TME). Biological pathway analysis confirmed that high-risk patients showed significant T- and B-cell immunosuppression and rapid tumor cell proliferation. More importantly, we researched the relationship between this IL(R)-based signature and immune checkpoints, tumor mutation burden (TMB), tumor purity and ploidy, and tumor immune dysfunction and exclusion (TIDE) score, which confirmed that this signature gave the best prognostic value. We first provided a robust prognostic IL(R)-based signature, which had the potential as a predictor for immunotherapy response to realize individualized treatment of LUAD.

## Introduction

Lung cancer is a major type of cancer and an important cause of cancer-related death in China and worldwide (1). Lung adenocarcinoma (LUAD) accounts for more than 40% of lung cancers and is also a major pathological subtype of lung cancer (2). Despite great advances in treatment strategies for lung cancer, including molecular targeted drugs and immune checkpoint inhibitors, the 5-year survival rate for lung cancer is only 17% (3). Therefore, it is necessary to find a method that can specifically predict patient survival so that the most appropriate personalized treatment can be tailored to different subgroups of patients with lung cancer. With the development of multi-omics, many studies using different expression profiles and bioinformatics have provided a variety of prognostic assessment methods for patients with LUAD. However, the parameters used in these studies were derived from genome-wide and transcriptome data and did not take into account the biological processes of the patients, which might lead to natural errors. In addition, these methods were simply mathematical models that might not reflect the intrinsic characteristics of the tumor itself.

With the successful application of immune checkpoint inhibitors (ICIs), the treatment effect of lung cancer has been significantly improved over the decades (4). Many studies have investigated the role of programmed cell death 1 (PD1)/programmed death-ligand 1 (PD-L1) in immunosuppression and verified their ability to act as a prognostic biomarker for tumor progression or as a biomarker for predicting immune response. However, these ICIs targeting PD-L1 and PD1 have a significant disadvantage that more than half of patients do not respond to PD-1/PD-L1 immunotherapy (5), suggesting the presence of other costimulatory signaling pathways in the tumor microenvironment of LUAD.

The IL(R) families refer to the lymphatic factors that interact between white blood cells or immune cells. They play an important role in transmitting information; activating and regulating immune cells; mediating the activation, proliferation, and differentiation of T or B cells; and regulating inflammatory response (6, 7). In the TME, tumor cells can produce a series of immunosuppressive factors, such as interleukin (IL)-10 or IL-4, which inhibit the activity of T cells and the killing ability of natural killer (NK) cells, and mediate the polarization of macrophages to the immunosuppressive direction (8, 9). Recently, some studies have shown that ILs could exert antitumor effects by enhancing the tumor therapeutic sensitivity of immune checkpoint inhibitors (10). Wen et al. showed that the IL20RA-mediated pathway formed a tumor-friendly immune microenvironment by increasing the expression of PD-L1 and reducing the recruitment of anticancer lymphocytes (11). IL-1β is secreted mainly by macrophages in immune response to pathogens. Inhibition or depletion of IL-1β in the TME has been verified to inhibit tumor vascular survival and various metastatic cell-induced lung metastases. Therefore, antibody strategy targeting the IL-1β signaling pathway showed great promise in curing lung cancer (12–14). Similarly, a variety of different ILs, such as IL-2, IL-15, IL-27, and IL-6, play an important role in tumor microenvironment and immunotherapy (13–17). In fact, many tumor therapies targeting IL-15 and IL-2 have shown positive therapeutic effects (15, 16). However, the expression profile and clinical features of IL(R) family in LUAD are still unclear.

We conducted a comprehensive analysis of the expression details and clinical features of IL(R) family in LUAD. In addition, 1,312 LUAD samples were selected from five public data sets to create and validate a prognostic model of five-IL(R)-based signature for LUAD. We first deeply analyzed the expression features and landscape of IL(R) family members in LUAD and validated an accurate IL(R)-based signature to serve as a reliable biomarker to predict the prognosis of LUAD. More importantly, based on comparison with other indicators for immunotherapy response, this five-IL(R)-based prognostic model showed a more powerful and reliable ability to predict the effect of immunotherapy and the prognosis of patients. Our findings will help clinicians implement individualized treatment for LUAD patients.

## Materials and Methods

### Publicly Data Collection

Cases with LUAD from five public databases were enrolled in this study. Among them, 464 LUAD samples with clinical characteristics were collected from The Cancer Genome Atlas (TCGA) (https://portal.gdc.cancer.gov/), which served as the training set. The other four independent validation sets containing 848 cases were downloaded from Gene Expression Omnibus (GEO) (http://www.ncbi.nlm.nih.gov/geo), including 117 samples from GSE13213, 85 samples from GSE30219, 226 samples from GSE31210, and 420 samples from GSE72094. Log2 conversion was performed for messenger RNA (mRNA) expression data, and the average expression amount was taken as the gene expression quantity. The basic clinical characteristics of these five cohorts are shown in **Table 1**.

**Table 1:** Clinical characteristics of lung adenocarcinoma from multiple cohorts.

Characteristics	TCGA cohort	GSE13213	GSE30219	GSE31210	GSE72094
	N = 464	N = 117	N = 85	N = 226	N = 420
Age	64.93 ± 0.4732	60.68 ± 0.94	61.49 ± 1.007	59.58 ± 0.4924	69.25 ± 0.4537
Gender					
Male	210 (45.3%)	60 (51.3%)	66 (77.6%)	105 (46.5%)	188 (44.8%)
Female	254 (54.7%)	57 (48.7%)	19 (22.4%)	121 (53.5%)	232 (55.2%)
Smoking					
Yes	386 (83.2%)	61 (52.1%)	/	111 (49.1%)	320 (76.2%)
No	66 (14.2%)	56 (47.9%)	/	115 (50.9%)	31 (7.4%)
NA	12 (2.6%)	0	/	0	69 (16.4%)
Stage					
I and II	358 (77.2%)	92 (78.6%)	84 (94.4%)	226 (100%)	334 (79.5%)
III and IV	98 (21.1%)	25 (21.4%)	1 (5.6%)	0	80 (19.1%)
NA	8 (1.7%)	0	0	0	6 (1.4%)
Status					
Alive	288 (62.1%)	68 (58.1%)	40 (47.1%)	191 (84.5%)	298 (71%)
Death	176 (37.9%)	49 (41.9%)	45 (52.9%)	35 (15.5%)	122 (29%)

### The Five IL(R)s Identification and Signature Generation

Based on TCGA transcriptome data, 87 IL(R)s were included in this study. Using R package “edge R”, 24 differently expressed genes (DEGs) were identified between normal and tumor tissues according to the standard of adjusted *p* < 0.001 and |log2 (fold change)| > 1. Univariate Cox regression analysis was used to analyze the relationship between the expression of IL(R)s and overall survival (OS) in LUAD, and seven IL(R)s were found to be associated with the prognosis of LUAD. Next, we performed a least absolute shrinkage and selection operator (LASSO) Cox regression model using R3.6.1 statistical software to figure out five IL(R)s (IL7R, IL5RA, IL20RB, IL11, and IL22RA1) that were thought to play the most important role in LUAD. A rigorous model-development process defined this five-IL(R)-based risk model, which was constructed by considering the expression of priority genes and the related risk coefficient as defined in the equation: risk score = −0.09948*IL7R + −0.51191*IL5RA + 0.09591*IL20RB + 0.28446*IL11 + 0.2596*IL22RA1. Patients with LUAD were divided into high- and low-risk groups based on the median value of risk score.

### Pathway and Function Enrichment Analysis

Kyoto Encyclopedia of Genes and Genomes (KEGG) and Gene Ontology (GO) pathway and functional enrichment analysis were performed using R statistical software and R packages.

### Analysis of Immune Cell Infiltration

CIBERSORT was used to estimate the abundance of immune cell infiltration in different risk groups in this study (17). CIBERSORT is a tool for deconvolution of the expression matrix of immune cell subtypes based on the principle of linear support vector regression, using RNA-seq data to estimate immune cell infiltration. In different tumors, this method of detecting the composition of immune cells is highly consistent with the real results (18). LM22 contains 547 genes that distinguish 22 human hematopoietic cell phenotypes, including seven T cell types, naive and memory B cells, plasma cells, NK cells, and myeloid subsets downloaded from the CIBERSORT web portal (https://cibersort.stanford.edu/) (17). CIBERSORT calculated the proportion of different immune cell types based on LM22 signature algorithm.

### GSVA and GSEA Analysis

The results of the seven metagenes clusters were emulated by Gene Sets Variation Analysis (GSVA), which evaluates whether a gene is highly or lowly expressed in sample in the context of the sample population distribution (19). Signaling pathways related to the IL(R)-based signature were analyzed through Gene Set Enrichment Analysis (GSEA). GSEA is commonly used to evaluate the distribution trend of genes in a predefined gene set, which has been widely reported to investigate the biological process difference between subtypes (20, 21).

### TMB and Neoantigen Analysis

Gene mutation data of patients with LUAD was generated from TCGA dataset (https://portal.gdc.cancer.gov/). The definition of tumor mutational burden (TMB) is mutations per million bases. The protein with specific amino acid sequence variation produced by cancer cells based on genetic variation is called “neoantigen”. We obtained neoantigen data of LUAD patients from The Cancer Immunome Atlas (TCIA) (https://tcia.at/home).

### TIDE and Immune Checkpoint Analysis

Tumor immune dysfunction and exclusion (TIDE) score was first defined by Jiang and his colleagues (22), which has been proven to have robust power for predicting the prognosis of cancer patients. We obtained TIDE score, IFN-γ (IFNG), merck18 (T-cell-inflamed signature) score, CD8 score, dysfunction score, and exclusion score from the TIDE web (http://tide.dfci.harvard.edu). The expression of immune checkpoints (PD-1, PD-L1, CTLA4, TIM-3, and LAG3) was extracted from TCGA database.

### Estimation of IDI and NRI

Net reclassification improvement (NRI) is often used to compare the accuracy of prediction ability of two models. To verify the improvement of the prognostic ability of the five-IL(R)-based signature, we estimated the integrated discrimination improvement (IDI) and NRI using R package of “PredictABEL”.

### Statistical Analysis

The patients with LUAD were divided into high- and low-risk groups according to median or optimal cutoff value. The Kaplan–Meier method was used to evaluate the OS between the high- and the low-risk group, and the log-rank was used to verify the significant difference. The unpaired *u*-test was used to analyze the distribution of immune cells, TMB, number of neoantigens, number of clonal neoantigens, number of subclonal neoantigens, PD-L1 protein expression, and TIDE in the different risk groups. Independent prognostic factors were calculated by Cox proportional hazard regression model. Among all the analysis methods, *p* < 0.05 was considered statistically different. R 3.6.1 (https://www.r-project.org) and GraphPad Prism 8.0.1. were used to analyze data and create tables and figures.

## Results

### Identification of Prognostic IL(R)s in LUAD

**Figure 1:**
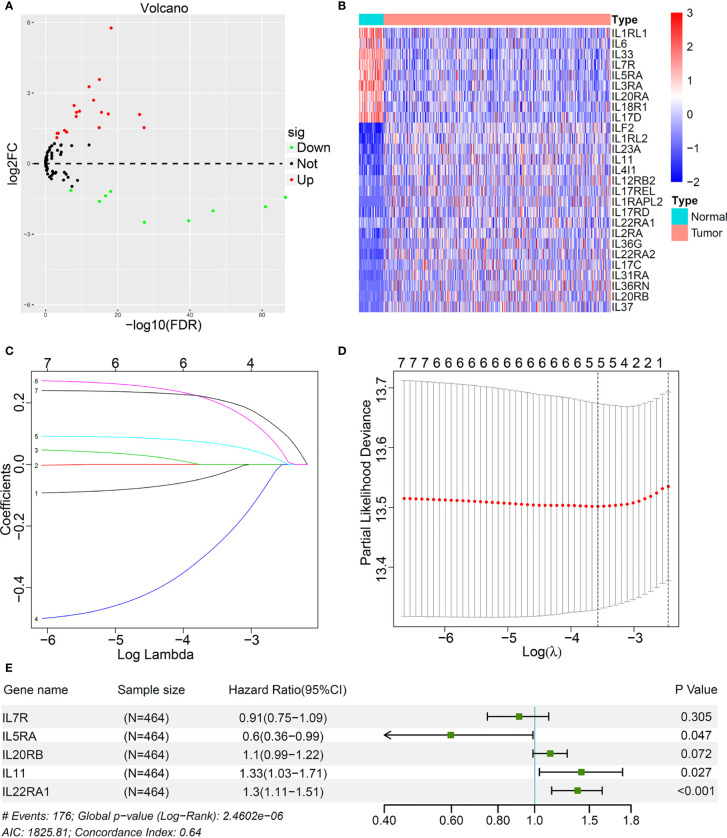
Identification of prognostic interleukins (IL) and interleukin receptors (ILR) in LUAD based on TCGA cohort. **(A)** The volcano map showed all IL(R) genes in LUAD comparing with normal tissues. **(B)** Heatmap showed 27 differentially expression genes (DEGs) panel. **(C)** LASSO coefficient profiles of the most useful prognostic genes. **(D)** 100-fold cross-validation for tuning parameter selection in the LASSO model. **(E)** Multivariable Cox proportional hazards regression analysis of the five prognostic genes.

Based on the standard of adjusted *p* < 0.001 and |log2 (fold change)| > 1), a total of 27 IL(R) family members with significant differences in LUAD were enrolled in this study (**Supplementary Table S1**). Volcano map (**Figure 1A**) and heatmap (**Figure 1B**) showed the expression characteristics of 27 DEGs. Univariate cox regression analysis for the 27 DEGs identified seven genes, which were significantly associated with OS (**Supplementary Table S2**). Five most important genes were further screen out using LASSO analysis (**Figures 1C, D**). Multivariable Cox analysis was performed to prove that IL5RA, IL11, and IL22RA1 were independent prognostic risk factors (p < 0.05) (**Figure 1E**).

### The Landscape and Prognostic Significance of the Five-IL(R)-Based Signature in LUAD

**Figure 2:**
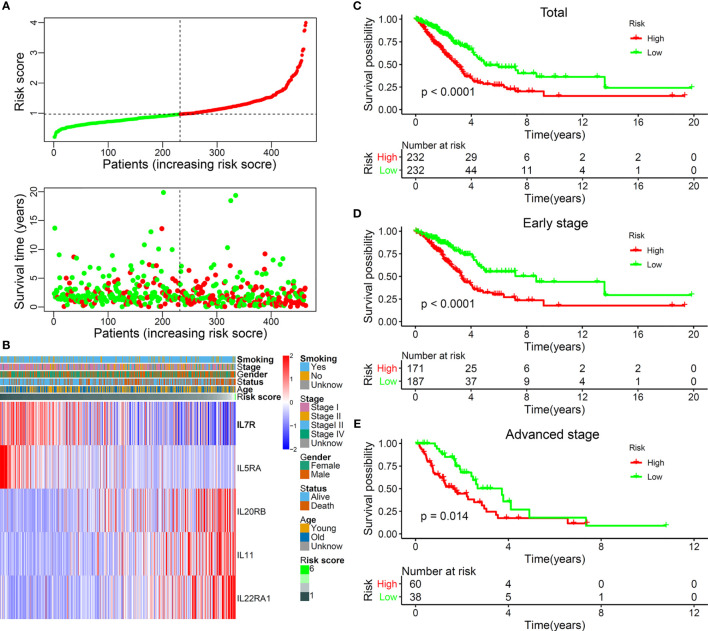
The landscape and prognostic significance of the five-IL(R)-based signature in LUAD using TCGA cohort. **(A)** the distribution of risk score and survival status. **(B)** Heatmap showed the expression characteristics of the identified ILs. **(C)** Kaplan-Meier curves compared the OS of total LUAD (n=464) between high- and low-risk groups. Kaplan-Meier curves compared the OS of early-stage (stage I and II) LUAD (n=358) **(D)** and advanced-stage (stage III and IV) LUAD (n=98) **(E)** between high- and low-risk groups.

A stepwise Cox proportional hazards regression model was constructed using the expression of the identified five IL(R)s and their corresponding regression coefficients: risk score= −0.09948*IL7R + −0.51191*IL5RA + 0.09591*IL20RB + 0.28446*IL11 + 0.2596*IL22RA1. All these patients were divided into high- and low-risk groups based on the median risk score. **Figure 2A** shows the distribution of survival status and risk score, which indicated that more deaths occurred in the high-risk group. **Figure 2B** exhibits the expression characteristics of these identified five IL(R)s. Patients with low risk score had high levels of IL7R and IL5RA. High expression of IL20RB, IL11, and IL22RA1 often occurred in patients with high risk score.

In order to verify the rationality of this five-IL(R)-based signature, we performed survival analysis on all cases and found that the OS of patients in the high-risk group was significantly lower than that in the low-risk group (**Figure 2C**, *p* < 0.0001). As known, lung cancer stage is an important factor in patient survival. There were significant differences between the treatment regimens in the early stage (stages I and II) and the advanced stage (stages III and IV) (23). Therefore, we analyzed the OS of patients in different stages and found that the OS of the high-risk group was significantly lower than that of the low-risk group, both in the early stage (**Figure 2D**, *p* < 0.0001) and the advanced stage (**Figure 2E**, *p* = 0.014).

### The Prognostic Power of the Five-IL(R)-Based in Clinical Subgroups

In order to further prove the powerful ability of this IL(R)-based signature to predict the prognosis of patients, we compared the OS of different risk groups in patients with different clinical subtypes (gender, age, and smoking history). The result confirmed that, in all clinical subgroups, patients with low risk score showed an obvious survival advantages (**Supplementary Figure S1**, *p* < 0.05).

Many factors can affect the OS of patients with lung cancer. Patients with different EGFR, KRAS, TP53, and STK11 mutation status were closely related to their prognosis and immunotherapy response (24, 25). In order to prove the powerful prognostic ability of this model, we compared the effects of high- and low-risk groups on OS in these gene mutation subgroups. Consistent with the expected result, the OS of high-risk group was significantly lower than that of low-risk group, no matter whether it was gene mutant or wild type (**Supplementary Figure S2**, *p* < 0.05).

### Validation of the Five-IL(R)-Based Signature in Four Other Independent Cohorts

**Figure 3:**
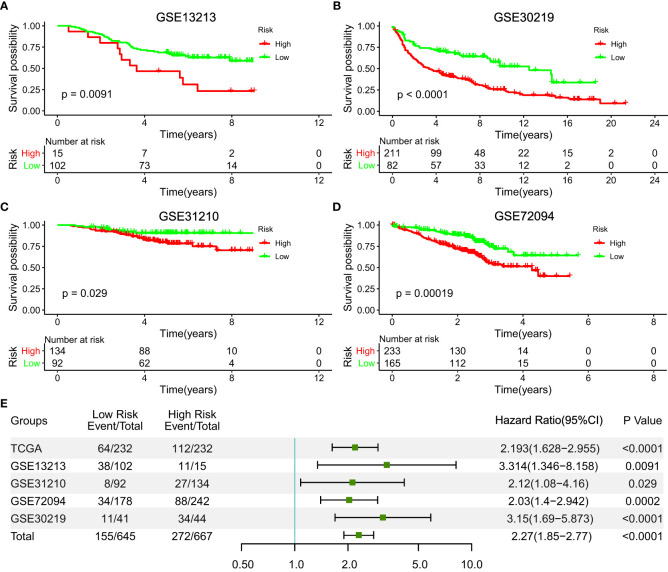
Validation of the prognostic value of the five-IL(R)-based signature in four independent GEO datasets. **(A)** GSE13213 (n=117); **(B)** GSE30219(n=85); **(C)** GSE31210 (n=226); **(D)** GSE72094 (n=420). **(E)** A meta-analysis based on prognostic results of the five independent datasets.

To verify the reproducibility of this five-IL(R)-based signature in LUAD patients, we first calculated risk values for each patient in four independent GEO datasets using the same formula. **Table 1** lists all demographic data for these public GEO datasets. Patients in different cohorts were divided into high- and low-risk groups based on optimal cutoff points. Not surprisingly, Kaplan–Meier analysis showed that patients in the high-risk group had a higher risk of death than those in the low-risk group, as shown in **Figure 3A** [hazard ratio (HR), 3.314; 95%CI, 1.346–8.158; *p* < 0.0001], **Figure 3B** (HR, 3.15; 95%CI, 1.69–5.873, *p <*0.0001], **Figure 3C** (HR, 2.12; 95%CI, 1.08–4.16; *p* = 0.029), **Figure 3D** (HR, 2.03; 95%CI, 1.4–2.942, *p* = 0.0002). In addition, we determined the prognostic significance of IL(R) family-based signatures in these public cohorts through a prognostic meta-analysis based on these five groups (n = 1,312). Our results confirmed that IL(R)-based signature was a risk factors for LUAD patients (HR, 2.028; 95%CI, 1.671–2.461, *p* < 0.0001) (**Figure 3E**).

### The Five-IL(R)-Based Signature Was an Independent Risk Factor for LUAD

In order to prove whether the predictive value of this five-IL(R)-based signature was affected by other clinical features, univariate and multivariate regression analysis was used, and the data showed that high risk score was an independent prognostic factor (HR, 1.724; 95%CI, 1.407–2.114, *p* < 0.0001). In addition, T and N stages were also independent prognostic factor (**Table 2**).

**Table 2:** Univariable and multivariable Cox regression analysis of the IL(R)-based signature in TCGA dataset.

Characteristics	Univariable analysis			Multivariable analysis		
	HR	95%CI	*p* value	HR	95%CI	*p* value
Age						
≤65 or >65	1.273	0.931–1.741	0.13			
Gender						
Female or male	0.837	0.613–1.143	0.262			
Smoking history						
Yes or No	0.964	0.619–1.502	0.872			
TNM stage						
Early stage or advanced stage	2.226	1.593–3.111	0	1.116	0.644–1.935	0.696
T stage						
1, 2, 3, or 4	1.565	1.287–1.902	0	1.356	1.095–1.679	0.005
N stage						
0, 1, 2, or 3	1.61	1.347–1.924	0	1.405	1.062–1.857	0.017
EGFR mutation						
Yes or no	1.311	0.86–1.997	0.208			
KRAS mutation						
Yes or no	1.068	0.736–1.55	0.728			
TP53 mutation						
Yes or no	1.175	0.86–1.606	0.31			
STK11 mutation						
Yes or No	0.926	0.585-1.468	0.745			
Risk score						
High or low	1.872	1.527–2.296	<0.0001	1.724	1.407–2.114	<0.0001

### Biological Pathways Related to the Five-IL(R)-Based Signature

**Figure 4:**
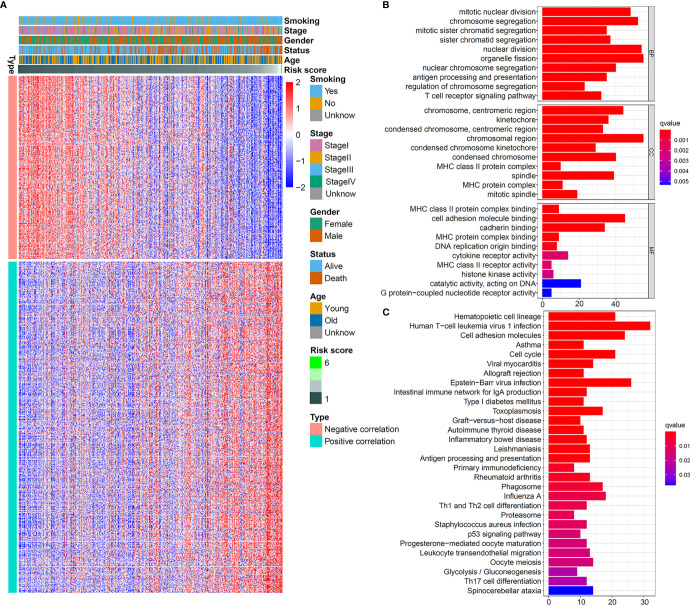
Biological pathways of the five-IL(R)-based signature in TCGA cohort. **(A)** Heatmap showed 262 genes most negatively correlated with IL -based risk score and 474 genes most positively correlated with IL(R)-based risk score in LUAD (Pearson |R| > 0.3, P<0.05). GO **(B)** and KEGG **(C)** analysis of the identified genes.

This powerful predictive ability of the five-IL(R)-based signature aroused our interest in exploring its potential mechanism. First of all, in order to be able to analyze the molecular biological characteristics of this model comprehensively, we screened out these genes strongly related to five-IL(R)-based signature score (Pearson |R| > 0.3, *p* < 0.05). The result indicated that 262 genes were negatively correlated with the risk score, and 474 genes were positively correlated with this IL(R)-based signature (**Figure 4A**). GO and KEGG function enrichment analysis was performed on the screened genes. As shown in **Figure 4B**, these genes were mainly involved in cell mitosis, proliferation, antigen processing and presentation, and immune regulation pathway (T-cell receptor signaling pathway, MHC-II protein signaling pathway, etc.). In addition, KEGG analysis showed that these genes were closely related to immune response, cell cycle, T-cell differentiation, p53 pathway, etc. (**Figure 4C**).

### The Immune Cell Infiltration Profile of the Five-IL(R)-Based Signature

Considering that the identified signature was closely related to immune-related pathways, we further analyzed the infiltration of immune cells in the high- and low-risk samples. The LM22 method in CIBERSORT was used to calculate the infiltration of immune cells in each TCGA sample. As shown in **Supplementary Figure S3A**, compared with LUAD patients in high-risk group, patients with low-risk score had higher proportion of B cells memory, T cells CD4 memory resting, monocytes, dendritic cells resting, and mast cells resting. However, macrophages M0, NK cells activated, and mast cells activated had a high proportion in the high-risk group. Specifically, memory B cells, resting memory CD4+ T cells, resting dendritic cells, resting mast cells, and monocytes were negatively correlated with risk score, whereas M0 macrophages, activated NK cells, activated mast cells, and follicular helper T cells were positively correlated with risk score (**Supplementary Figure S3B**). **Supplementary Figure S3C** exhibits the distribution of the main immune cell populations in the two risk groups. M0 macrophages and resting memory CD4+ T cells were the main components of the tumor immune infiltrate in patients at both high and low risk.

### Inflammatory and Immunologic Profile of the Five-IL(R)-Based Signature

**Figure 5:**
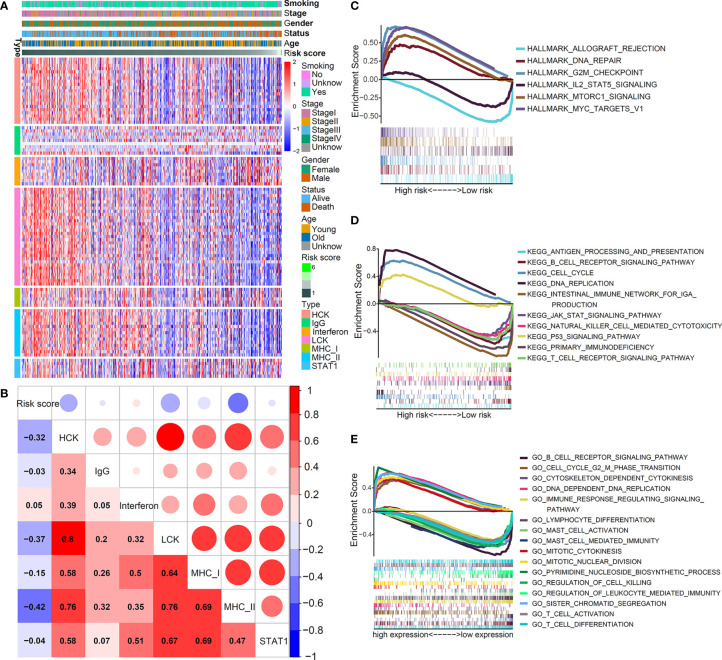
Inflammatory and immunologic profile of the five-IL(R)-based signature in TCGA cohort. **(A)** Heatmap showed the relationship between risk score and immune inflammatory metagenes. **(B)** Correlogram was generated based on Pearson r-value between risk score and metagenes. **(C–E)** The different gene sets enrichment analysis based on GSEA.

To further understand the inflammatory profile associated with this IL(R)-based signature, we investigated the relationship between risk score and seven metagenes (**Figure 5A**). We used GSVA to simulate the corresponding results of seven metagenes (22) and found that the risk score was negatively correlated with MHC-II, HCK, and LCK (**Figure 5B**).

Although previous studies have shown that this signature was related to immunity, the enrichment of antitumor and tumor-promoting pathways in the TME of different risk groups was not clear. Then, we performed GSEA enrichment analysis. As shown in **Figure 5C**, hallmark analysis showed that the tumor-promoting pathways—DNA_REPAIR, G2M_CHECKPOINT, MTORC1_SIGNALING, and MYC_TARGETS_V1—were enriched in high-risk group, while the antitumor pathways—ALLOGRAFT_REJECTION and IL2_STAT5_SIGNALING—were mainly enriched in low-risk group. The KEGG analysis indicated that CELL_CYCLE, DNA_REPLICATION, and P53_SIGNALING_PATHWAY that promoted cell proliferation were mainly enriched in the high-risk group, while multiple T cell-, B cell-, and NK cells-mediated immune pathways were activated in the low-risk group (**Figure 5D**). The GO analysis yielded similar results, which further verified that cell proliferation signaling pathways in the high-risk group were obviously activated, and tumor immune-related pathways mediated by T and B cells were significantly enhanced in the low-risk group (**Figure 5E**). **Supplementary Table S3** shows the enrichment score (NES) and nominal *p* value. These results fully explained the reason that the prognosis of patients in the high-risk group was worse than that in the low-risk group from the perspective of molecular biology.

### Relationship Between the Five-IL(R)-Based Signature and Immunotherapy-Related Biomarkers

**Figure 6:**
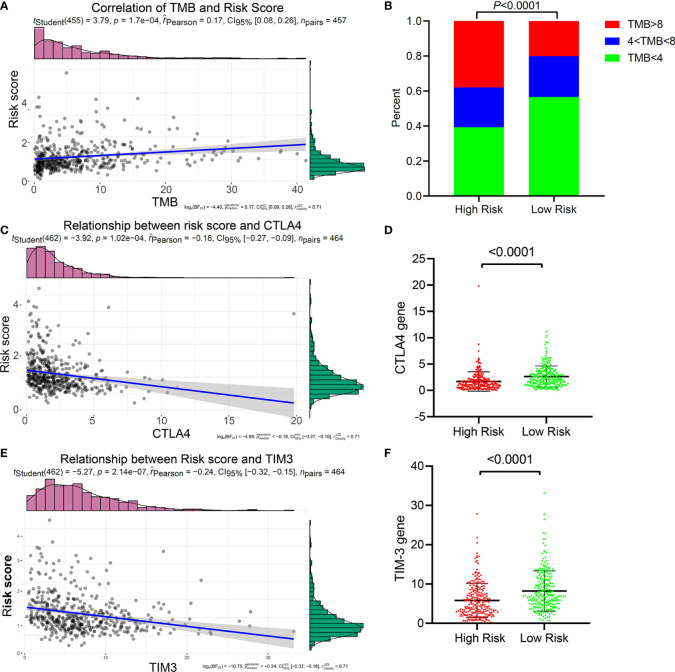
Relationship between the five-IL(R)-based signature and immunotherapy-related biomarkers in TCGA cohort. **(A)** Correlation of TMB and risk score. **(B)** The proportion of TMB in the high-risk group and the low-risk groups. **(C)** Correlation of CTLA4 and risk score. **(D)** Correlation of TIM3 and risk score. **(E)** Comparison of CTLA4 in high-risk group and low-risk group. **(F)** Comparison of TIM3 in high-risk group and low-risk group.

Immunotherapy targeting immune checkpoints has now become the first-line treatment of lung cancer, especially advanced tumors. At present, PD1, PD-L1, TMB, LAG3, CTLA4, and TIM3 have been widely used as biomarkers of immunotherapy response (26). Studies have shown that patients with high TMB have better treatment outcomes with ICIs (27). We investigated the relationship between the five-IL(R)-based signature and these immunotherapy biomarkers and found that patients with high-risk score tended to have higher TMB (**Supplementary Figure S4**). Correlation analysis showed that risk score was positively correlated with TMB (**Figure 6A**). Although lung cancer patients with high TMB had better immunotherapy response and prognosis, a study has confirmed that postoperative adjuvant chemotherapy for patients with TMB <4 can significantly improve the survival rate (28). Using the same cutoff value, we found that the proportion of patients with TMB <4 in the low-risk group was significantly higher than that in the high-risk group. At the same time, the proportion of patients with TMB >8 in the low-risk group was significantly lower than that in the high-risk group (**Figure 6B**). These results suggested that low-risk patients were more likely to benefit from chemotherapy, while high-risk patients were more likely to benefit from immunotherapy. Of course, more clinical cohort studies are needed to verify this conclusion.

In order to prove whether this five-IL(R)-based risk score can be used as a basis for LUAD patients to receive ICI therapy, we have deeply explored the relationship between the risk score and the current major immune checkpoints (PD1, PD-L1, LAG3, CTLA-4, and TIM-3). The results confirmed that the risk score had a strong negative correlation with CTLA-4 (**Figure 6C**) and TIM-3 (**Figure 6D**), and the expression of CTLA-4 (**Figure 6E**) and TIM-3 (**Figure 6F**) in the low-risk group were significantly higher than those in the high-risk group. Interestingly, this risk score has no relationship with the expression levels of the other three immune checkpoints (PD1, PDL1, and LAG3) (**Supplementary Figure S5**). Considering that patients with high expression of CTLA-4 was more likely to benefit from immunotherapy of anti-CTLA-4, and that anti-TIM-3 or anti-CTLA-4 could enhance tumor immunity, we speculated that patients in the low-risk group may be more sensitive to CTLA-4 and TIM-3 inhibitors.

### Distribution of Number of Tumor Neoantigens and Tumor Purity or Ploidy in the Five-IL(R)-Based Signature

**Figure 7:**
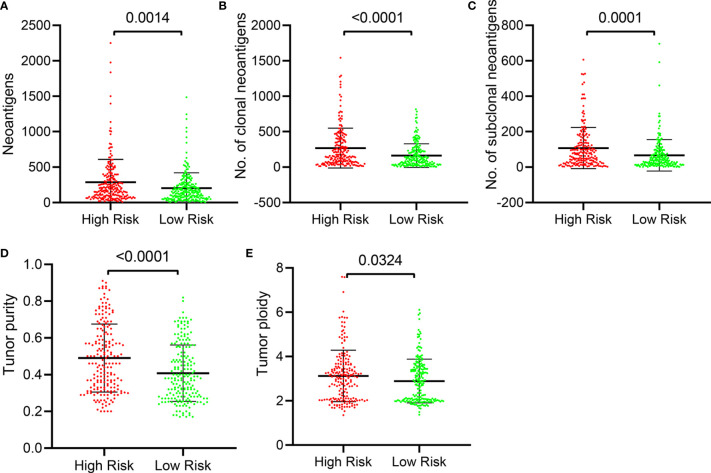
Distribution of number of tumor neoantigens and purity in the five-IL(R)-based signature in TCGA cohort. The distribution and comparison of number of neoantigens **(A)**, number of clonal **(B)**, number of subclonal **(C)**, Tumor purity **(D)**, and tumor ploidy **(E)** in the high-risk group and the low-risk group. Tumor purity, the proportion of cancer cells in a tumor sample; tumor ploidy, the true content of cancer cells in a tumor sample caused by abnormal chromosomal structure and number.

Neoantigen is a protein encoded by a mutated gene in tumor cells. Corresponding to different mutations, these neoantigens also exhibit intratumoral heterogeneity. Neoantigens are potential biomarkers for predicting patient response to immunotherapy, and the distinction between clonal and subclonal neoantigens can also help identify which neoantigens are most effective and can develop different targeting methods (29). Clonal neoantigens exist in every cancer cell, while subclonal neoantigens are expressed only in part of cancer cells. Clonal and subclonal events in cancer evolution have a profound impact on tumor therapy (30). We explored the relationship between tumor neoantigens and this five-IL(R)-based signature. Our results showed that the number of neoantigens (**Figure 7A**), the number of clonal neoantigens (**Figure 7B**), and the number of subclonal neoantigens (**Figure 7C**) were higher in high-risk group. Tumor purity refers to the proportion of cancer cells in a tumor sample, while tumor ploidy refers to the true content of cancer cells in a tumor sample caused by abnormal chromosomal structure and number. Studies have shown that tumor purity was a key factor in the prognosis of patients (31). We further evaluated the relationship between risk score and tumor purity and ploidy, which showed that the tumor purity and ploidy in high-risk patients were higher than that in low-risk group (**Figures 7D, E**).

### Relationship Between the Five-IL(R)-Based Signature and Tumor Immune Dysfunction and Exclusion Score

**Figure 8:**
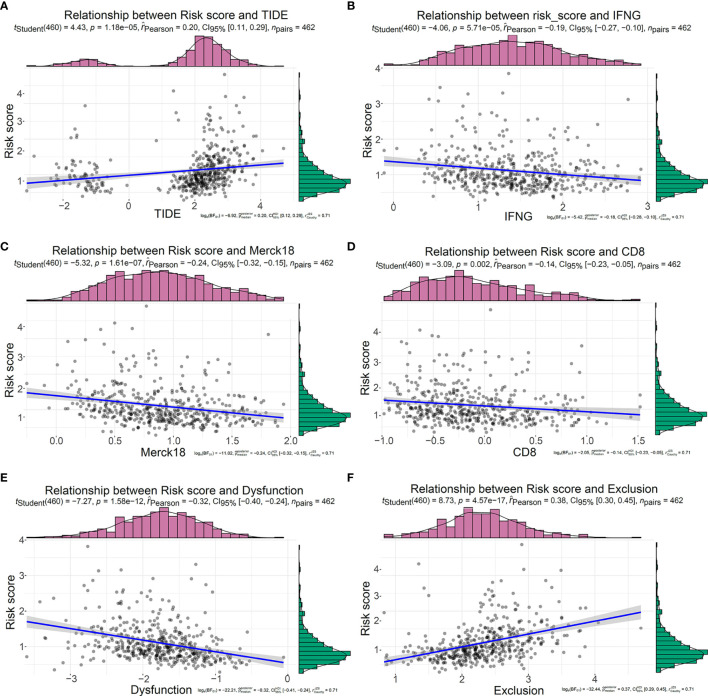
Relationship between the five-IL(R)-based signature and tumor immune dysfunction and exclusion score in TCGA cohort. Correlation analysis between risk score and TIDE **(A)**, IFNG **(B)**, Merck18 **(C)**, CD8 **(D)**, T cell Dysfunction **(E)**, T cell Exclusion **(F)**.

TIDE, a more accurate biomarker than TMB and ICIs, is a computational approach that simulates the two main mechanisms of tumor immune escape: induction of T cell dysfunction in tumors with high cytotoxic T lymphocyte (CTL) invasion and prevention of T cell invasion in tumors with low CTL levels (22). Although cytotoxic T cells can infiltrate some tumors, they are still unable to inhibit tumor growth in a T-cell dysfunctional state. T-cell exclusion was presented in tumors with low T-cell invasion. Several molecular mechanisms may explain the lack of T-cell infiltration in tumors, such as impaired initiation of tumor-specific T cells or the presence of suppressor cells that prevent T-cell infiltration into tumors (23). It was reported that T-cell-inflamed phenotype could predict response to pembrolizumab in multiple tumor types (32). Calculated based on the expression level of genes in a specific gene set, T cell dysfunction score and T cell exclusion score were reported to have well-prediction performance for ICB response (22). Here, TIDE score, T-cell-inflamed signature (merck18), T-cell dysfunction score, T-cell exclusion score, IFNG score, and CD8 were generated from TIDE system. In order to further study the value of this model in tumor immunotherapy, we explored the relationship between this risk signature and TIDE. In our study, the risk score was positively correlated with TIDE score (**Figure 8A**) and T-cell exclusion score (**Figure 8F**) but negatively correlated with IFNG (**Figure 8B**), merck18 score (**Figure 8C**), CD8 (**Figure 8D**), and T-cell dysfunction score (**Figure 8E**). Compared with low-risk patients, high-risk patients had higher TIDE score (**Supplementary Figure S6A**) and T-cell exclusion score (**Supplementary Figure S6F**), while they had lower level of IFNG (**Supplementary Figure S6B**), merck18 score (**Supplementary Figure S6C**), CD8 (**Supplementary Figure S6D**), and T-cell dysfunction score (**Supplementary Figure S6E**).

### Comparison of the Prognostic Power of the Five-IL(R)-Based Signature With Other Biomarkers

**Figure 9:**
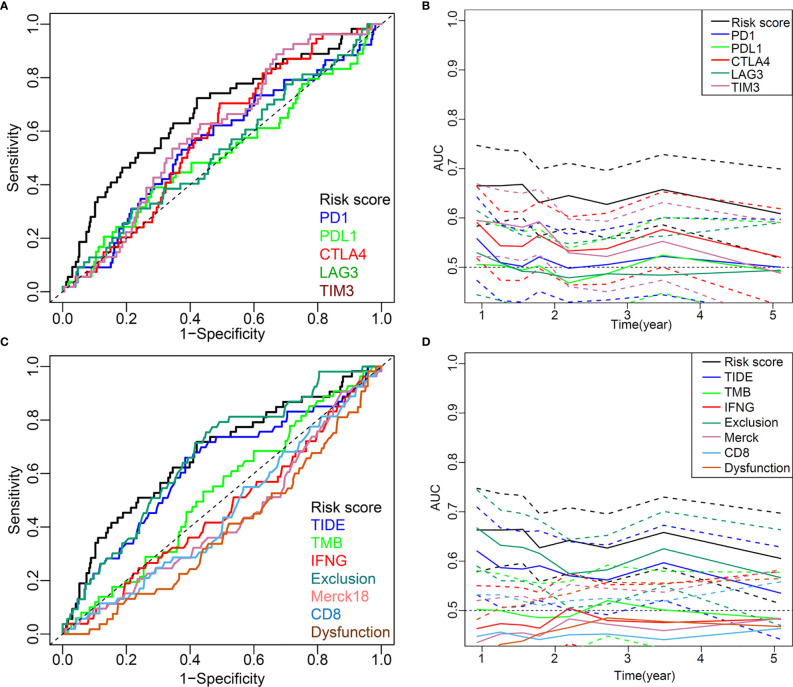
Comparison of the predictive power of this five-IL(R)-based signature with other biomarkers in TCGA cohort. **(A, C)** ROC curve compared the sensitivity and specificity of risk score and other markers for predicting OS. **(B, D)** Time-dependent AUC reflected and compared the predictive power of risk score and other markers on OS. The dotted lines represented the 95% confidence interval.

The five-IL(R)-based signature was closely associated with other immunotherapy-related biomarkers, and it was also an independent risk factor for OS in patients (**Table 2**). To confirm the advantages of the model in predicting the prognosis of lung cancer, we compared this signature with other markers by receiver operating characteristic (ROC) analysis (**Figures 9A, C**). The time-dependent AUC showed that the prognostic power of the IL(R)-based signature was significantly higher than that of classical immunotherapy markers, including PD1, PD-L1, TMB, and CTLA4, even the newly discovered biomarker, TIDE (**Figures 9B, D**). Similar to ROC, net reclassification improvement (NRI) and integrated discrimination improvement (IDI) are used to compare the predictive power of two indicators or models (33). In this study, the NRI, and IDI were further used to compare the accuracy between the five-IL(R)-based signature and other markers. As is shown in **Table 3**, the prediction performance of this signature was better than TMB, TIDE score, IFNG, merck18, CD8, T-cells dysfunction and exclusion, PD-1, PD-L1, CTLA-4, LAG3, and TIM-3 (NRI > 0, *p <*0.05).

**Table 3:** Compare the predictive value and predictive power of the risk score model with other indicators using NRI and IDI.

Indicators (Compared by risk score)	NRI (continuous) (95%CI)	*p* value	IDI (95%CI)	*p* value
TMB	0.2426 (0.0591–0.426)	0.0096	0.0538 (0.0301–0.0776)	<0.0001
TIDE score	0.301 (0.1183–0.4838)	0.0013	0.0534 (0.0309–0.076)	<0.0001
IFNG	0.3164 (0.132–0.5009)	0.0008	0.0494 (0.0264–0.0724)	<0.0001
Merck18	0.3126 (0.1297–0.4955)	0.0008	0.0483 (0.0256–0.0711)	<0.0001
CD8	0.3126 (0.1297–0.4955)	0.0008	0.0507 (0.0276–0.0737)	<0.0001
Dysfunction	0.319 (0.135–0.503)	0.0007	0.0457 (0.0237–0.0676)	<0.0001
Exclusion	0.0749 (-0.1126–0.2624)	0.4338	0.0255 (0.0023–0.0487)	0.03129
PD1	0.2719 (0.0918–0.4519)	0.0031	0.0547 (0.0319–0.0774)	<0.0001
PDL1	0.2456 (0.0638–0.4275)	0.0081	0.0556 (0.0328–0.0785)	<0.0001
CTLA4	0.1932 (0.0085–0.3779)	0.0404	0.0341 (0.0102–0.0579)	0.0051
LAG3	0.3121 (0.1302–0.4939)	0.0008	0.0522 (0.0289–0.0755)	<0.0001
TIM3	0.3121 (0.1276–0.4966)	0.0009	0.0445 (0.0217–0.0672)	0.0001

## Discussion

With the development of high-throughput sequencing technology in the past few years, increasing prognostic markers and immunotherapy targets have been discovered, which can help us better understand tumors. However, presently, there is still no biomarker that could accurately reflect the immunotherapy response and prognosis of LUAD. They could not truly reflect the characteristics of the tumor microenvironment. Therefore, we proposed the IL(R)-based signature to predict the prognosis of LUAD for the first time, which was the first comprehensive understanding of the prognostic characteristics of IL(R) families and their prognostic effect on immunotherapy. First of all, this five-IL(R)-based signature demonstrated its powerful and reliable prognostic ability under the verification of five independent cohorts containing 1,312 cases. Second, through immune microenvironment and signal pathway analysis, we found that the strong prognostic ability of this IL(R)-based signature was attributed to unique immune cell infiltration ratio, tumor cell proliferation activity, immune cell activity, and antigen processing and presentation mediated by MHC I and MHC II in different risk groups. In addition, we deeply analyzed the potential value of this signature as a biomarker of tumor immunotherapy response and found that low-risk patients were more likely to benefit from postoperative chemotherapy, and they also might be benefit from ICI therapy based on anti-CTLA-4 or anti-TIM-3. More importantly, after comparing with other classic predictors for immunotherapy response, we found that this five-IL(R)-based signature had better prediction performance than other indicators. This study gave us a comprehensive understanding of the role of IL(R)s in LUAD. The classification method based on this signature will help clinicians better implement individualized treatment for patients with LUAD.

Although the IL(R)-based signature exhibited powerful predictive ability, these signature members themselves (IL-7R, IL-5RA, IL-20RB, IL-11, and IL-22RA1) were rarely reported to be used to predict tumor prognosis. It was reported that IL-11 played an important role as a prognostic factor in multiple tumors (34–36). Interestingly, IL-5, IL-7, IL-20, and IL-22, rather than their receptors, have been widely reported to have the ability to predict tumor prognosis (37–39).

Tumors have the ability to form their microenvironment to counteract the host immune system, and one of the key challenges of tumor immunotherapy is to overcome tumor-induced immunosuppression. Immune cells of the innate immune system and adaptive immune system constitute the main components of TME. IL(R)s are key mediators of cell–cell interaction in TME and have the function of activating lymphocytes. IL-7R is a heterodimer composed of IL-7Rα and common γ chain, which can combine with various ILs, including IL-2, IL-4, IL-7, IL-9, IL-15, and IL-21 (40). Over 70% blasts with T-cell acute lymphoblastic leukemia (T-ALL) patients showed IL-7R-positive expression (41). IL-7R could inhibit apoptosis in T-ALL blasts by binding with IL-7 (41). IL-7R signal transduction has been shown to be associated with the prognosis of malignant lymphoma. Specifically, a gain-of-function mutation in IL-7R played an oncogene role in approximately 10% of T-cell ALLs and 1% of B-cell ALLs (42). Therefore, anti-IL-7R targeting antibody therapies had the potential to be beneficial for the patients with lymphoid malignancy (43). Human IL-5R alpha-chain (IL-5RA) was a soluble form of IL-5R that contained the extracellular IL-5 binding domain affecting the activation of human eosinophils and basophils (44). IL-5RA intracellular signaling provoked eosinophils proliferation and exaggerated activation through FIP1L1-PDGFRA/JAK2/Lyn/Akt network complex, which manifested as chronic eosinophilic leukemia (CEL) (45). IL-20RB forms a heterodimer structure with IL-20RA or IL-22R1, which is highly expressed in skin cells, lung, and reproductive organs. Targeted binding with IL-20 or IL-10 family members could induce cell proliferation (46, 47). Anne et al. found that IL-20 and its receptors were often maladjusted in NSCLC, and IL-20RB mRNA was significantly increased in NSCLC. Targeting this family members may be a viable therapeutic option in lung cancer (48). IL-11 is a member of glycoprotein 130 (GP-130) cytokines and participates in the GP-130 signaling pathway with other cytokines of the same family. IL-11 was identified in a series of cells, including T cells, B cells, and macrophages, but the main source of IL-11 secretion remains unclear (49). There were a number of reports that documented the involvement of IL-11 in various malignancies including gastric, colorectal, pancreatic, prostate, breast, ovarian, endometrial, and bone cancers, and some studies have been published on targeting IL-11 signaling in preclinical models of cancer (50). Interleukin-20 receptor subunit alpha (IL20RA) belongs to the type II cytokine receptor family. Upon binding to its ligands, such as IL-19, IL-20, and IL-24, IL20RA can form a functional heterodimeric receptor with IL20RB. IL20RA promoted stem cell characteristics and tumor initiation ability of breast cancer cells through JAK1–STAT3–SOX2 signaling pathway, resulting in increased expression of PD-L1 and reduced recruitment of lymphocytes, including CD8 T cells and NK cells, so as to form a tumor-favorable immune microenvironment (11). Although these reports indicated the potential application of targeting this five IL(R)s in cancer treatment, further research will be necessary to assess their value in LUAD.

In our study, 87 IL(R)s extracted from TCGA database were included. After difference analysis, univariate Cox regression analysis, LASSO regression analysis, and multivaritate Cox regression analysis, we finally constructed a prognostic model for LUAD based on five IL(R)s. GO and KEGG analysis of 736 genes that strongly correlated with the five-IL(R)-based signature showed that this model is mainly related to cell mitosis, cell cycle, proliferation, antigen processing and presentation, immune regulation pathway, immune response-related diseases, T-cell differentiation, or p53 pathway. GSVA and GSEA analysis comforted that the signal pathways promoting cell proliferation were activated in the high-risk group, while the adaptive immune response seemed suppressed. In contrast, T (and B)-cell-mediated tumor immunity appeared significantly enhanced in the low-risk group. By describing the gene expression profile associated with the five-IL(R)-based signature, these results illustrated the biological processes predicting the prognosis of LUAD.

The role of IFNG in immunotherapy response remains controversial (51). On the one hand, IFNG was known to play a key role in antitumor immunity. Interferon played an important role in the early stage of antigen recognition and the interaction between adaptive immune cells and innate immune cells. Therefore, the loss of functional mutations and genomic changes in IFN signaling pathway were associated with clinical immune checkpoint blockade (ICB) resistance or recurrence (52, 53). On the other hand, activation of IFNG signaling pathway in tumor cells can antagonize the function of T cells and innate immune cells. Blocking of IFNG signaling pathway in tumor cells can improve the body’s ability to kill tumor cells and promote the response to ICB (51). CD8+ T cells are the main effector cells that carry out antigen-specific killing of tumor cells. Effectively enhancing the antitumor function of CD8+ T cells is the key to the treatment of tumors (54, 55). CD8+ T cells could be used as a marker of immunotherapy response. Based on the above results, we further demonstrated the potential of the risk model serving as indicators for immunotherapy response. Currently, the most recognized biomarkers (PD-1, PD-L1, CTLA-4, TIM-3, and LAG3) still cannot accurately guide the use of ICIs, resulting in limited clinical benefit for cancer patients (26). TMB, neoantigens, and TIDE were newly discovered immunotherapy predictors (26). Especially TIDE has been proven to have better prognostic performance than other biomarkers or indicators (22). To prove that this signature can be a biomarker of immunotherapy response, we explored the relationship between this signature and other markers mentioned above. The results showed that the proportion of patients with TMB <4 in low-risk group was significantly higher than that in high-risk group, and the expression levels of CTLA-4 and TIM-3 in low-risk patients were significantly higher than those in high-risk patients. Considering that patients with high expression of CTLA-4 (56) or TIM-3 (57) had a better prognosis, inhibition of CTLA-4 or TIM-3 expression could enhance tumor immunity (58, 59), and postoperative adjuvant chemotherapy for patients with TMB <4 can significantly improve the survival rate (28), we speculated that patients in the low-risk group may be more likely to benefit from chemotherapy and more sensitive to CTLA-4 and TIM-3 inhibitors.

It was confirmed that higher TIDE score was less likely to benefit from anti-PD-1/CTLA-4 (22), and merck18 (T-cell-inflamed signature) can contribute to T-cell dysfunction (60). Hence, this IL(R)-based signature identified low-risk patients who should be suitable for treatment with ICIs for their lower TIDE score and T-cell dysfunction score. To verify the superiority of this signature, we compared the prognostic power of this five-IL(R)-based signature with other indicators. The ROC and time-dependent area under the curve (AUC) values showed that our signature got a better prediction performance than other markers. In addition, the NRI and IDI analysis also verified that this five-IL(R)-based signature was superior to other markers.

Although the IL(R)-based signature can be used as an effective independent prognostic factor and can predict the immunotherapy response in LUAD, this study still had some limitations. First of all, all these five cohorts were retrospective datasets, and a prospective study of this IL(R)-based signature will be necessary. Second, all the expression data were sequencing data downloaded from public database, and the findings will need to be validated by new method and fresh specimens. Third, the ability to predict the immunotherapy response was evaluated indirectly, and further research is needed to verify this finding.

In summary, our study thoroughly described the overall expression profile and clinical characteristics of the five-IL(R)-based signature in LUAD and provided more information about the immune microenvironment and immunotherapy response. This was the first time to propose the prognostic model based on IL(R) family members, which provided a marker for precisely predicting the prognosis of LUAD. In addition, this research indirectly proved the possibility of this IL(R)-based signature serving as indicator for tumor immunotherapy response, which will provide important guidance for clinicians to achieve individualized treatment for patients with LUAD.

## Data Availability Statement

The original contributions presented in the study are included in the article/**Supplementary Material**. Further inquiries can be directed to the corresponding author.

## Author Contributions

TF and SY designed the experiment. HB and TF analyzed the data. LZ and DL interpreted the data. SP and TF wrote the manuscript. QG carefully reviewed the manuscript. All authors contributed to the article and approved the submitted version.

## Funding

This work was supported by the National Natural Science Foundation of China (81700093, 81770095), and Hubei Provincial Natural Science Foundation of China (2020CFA027).

## Conflict of Interest

The authors declare that the research was conducted in the absence of any commercial or financial relationships that could be construed as a potential conflict of interest.

## Publisher’s Note

All claims expressed in this article are solely those of the authors and do not necessarily represent those of their affiliated organizations, or those of the publisher, the editors and the reviewers. Any product that may be evaluated in this article, or claim that may be made by its manufacturer, is not guaranteed or endorsed by the publisher.
