# Supplementary material for: Clinical Significance and Immunologic Landscape of a Five-IL(R)-Based Signature in Lung Adenocarcinoma
Source: Front Immunol. 2021 Aug 23;12:693062. doi: 10.3389/fimmu.2021.693062 (PMC8419226; doi:10.3389/fimmu.2021.693062)
Supplement: Supplementary file 1 [file DataSheet_1.docx]

| **Supplementary Table S1. Distinct interleukin family genes** | | | | |
| --- | --- | --- | --- | --- |
| Gene | logFC | logCPM | PValue | FDR |
| IL3RA | -1.431834147 | 14.20297295 | 3.90E-69 | 3.40E-67 |
| IL7R | -1.831356272 | 14.72280479 | 2.84E-63 | 1.24E-61 |
| IL33 | -2.006549593 | 14.57004851 | 1.39E-48 | 4.04E-47 |
| IL6 | -2.42905454 | 13.96267477 | 1.15E-41 | 2.49E-40 |
| IL1RL1 | -2.492009191 | 12.99459052 | 2.34E-29 | 4.08E-28 |
| ILF2 | 1.531766532 | 17.84558587 | 3.64E-29 | 5.28E-28 |
| IL4I1 | 2.087519209 | 14.03239835 | 7.49E-28 | 9.31E-27 |
| IL37 | 5.761748908 | 14.72614545 | 6.48E-20 | 7.05E-19 |
| IL18R1 | -1.181463483 | 12.7178235 | 9.05E-20 | 8.75E-19 |
| IL1RL2 | 2.109726301 | 12.66390331 | 5.11E-19 | 4.45E-18 |
| IL20RA | -1.36688302 | 13.05412527 | 3.18E-18 | 2.51E-17 |
| IL23A | 2.181546786 | 13.07675464 | 4.70E-17 | 3.41E-16 |
| IL5RA | -1.608035958 | 12.18715591 | 1.73E-16 | 1.16E-15 |
| IL20RB | 3.576000234 | 13.49084147 | 1.92E-16 | 1.19E-15 |
| IL2RA | 1.529414113 | 13.29339201 | 2.46E-16 | 1.43E-15 |
| IL31RA | 2.696072626 | 12.43218385 | 9.26E-15 | 5.04E-14 |
| IL36RN | 3.26929202 | 12.52195758 | 1.94E-13 | 9.39E-13 |
| IL22RA2 | 2.226802088 | 12.24172569 | 8.92E-11 | 4.08E-10 |
| IL36G | 2.178891616 | 12.21763325 | 6.83E-10 | 2.83E-09 |
| IL11 | 2.005664276 | 12.3286931 | 8.48E-10 | 3.36E-09 |
| IL17C | 2.470384672 | 12.31126095 | 3.46E-09 | 1.31E-08 |
| IL17D | -1.141255633 | 12.24969578 | 2.92E-08 | 9.78E-08 |
| IL17RD | 1.340486258 | 12.4799409 | 4.50E-07 | 1.40E-06 |
| IL22RA1 | 1.412966631 | 12.6862884 | 1.95E-06 | 5.66E-06 |
| IL1RAPL2 | 1.288674135 | 12.02744817 | 0.000123784 | 0.00029106 |
| IL17REL | 1.288148395 | 12.0887217 | 0.000278628 | 0.000621555 |
| IL12RB2 | 1.104504818 | 12.14293302 | 0.000346891 | 0.000754488 |

| **Supplementary Table S2. Univariable analysis for differently expressed genes(DEGs)** | | |
| --- | --- | --- |
| DEGs | HR | P |
| IL22RA1 | 1.337768797 | 2.56E-05 |
| IL11 | 1.517573621 | 0.000352827 |
| IL20RB | 1.189997186 | 0.000422479 |
| IL7R | 0.78458044 | 0.009656394 |
| IL5RA | 0.564113951 | 0.023159841 |
| IL33 | 0.864158846 | 0.03444645 |
| IL20RA | 1.182960866 | 0.049383797 |
| IL3RA | 0.770813437 | 0.063960936 |
| IL18R1 | 1.309009835 | 0.098146575 |
| IL22RA2 | 0.778605995 | 0.121670997 |
| ILF2 | 1.1118409 | 0.226618096 |
| IL23A | 1.102684898 | 0.248241993 |
| IL17RD | 1.128348446 | 0.328635852 |
| IL17D | 0.824699456 | 0.358812161 |
| IL1RL2 | 1.118031921 | 0.362973743 |
| IL17C | 1.082413996 | 0.459197014 |
| IL1RAPL2 | 0.846919653 | 0.505415305 |
| IL4I1 | 1.054478925 | 0.525855998 |
| IL36RN | 1.038845758 | 0.625822919 |
| IL12RB2 | 1.075250677 | 0.677005416 |
| IL17REL | 0.960828631 | 0.838300333 |
| IL37 | 0.991769528 | 0.843400679 |
| IL36G | 0.98106857 | 0.886012717 |
| IL6 | 1.004027117 | 0.94943735 |
| IL31RA | 1.003138165 | 0.973938682 |
| IL2RA | 0.996574015 | 0.974885349 |
| IL1RL1 | 0.997890801 | 0.980446231 |

| **Supplementary Table S3: GSEA analysis based on the risk score.** |  |  |  |
| --- | --- | --- | --- |
| [NAME](http://www.gsea-msigdb.org/gsea/msigdb/cards/KEGG_INTESTINAL_IMMUNE_NETWORK_FOR_IGA_PRODUCTION) | NES | NOM p-val | FDR q-val |
| KEGG_CELL_CYCLE | 2.0944207 | 0 | 0.016460106 |
| KEGG_DNA_REPLICATION | 1.9583435 | 0 | 0.04008902 |
| KEGG_P53_SIGNALING_PATHWAY | 1.6384557 | 0.01629328 | 0.16592142 |
| KEGG_INTESTINAL_IMMUNE_NETWORK_FOR_IGA_PRODUCTION | -2.134149 | 0.002028398 | 0.004547886 |
| KEGG_B_CELL_RECEPTOR_SIGNALING_PATHWAY | -2.066619 | 0.002096436 | 0.004330311 |
| KEGG_T_CELL_RECEPTOR_SIGNALING_PATHWAY | -1.922312 | 0.004032258 | 0.016309438 |
| KEGG_JAK_STAT_SIGNALING_PATHWAY | -1.7974933 | 0.002070393 | 0.033968464 |
| KEGG_NATURAL_KILLER_CELL_MEDIATED_CYTOTOXICITY | -1.7772962 | 0.012195122 | 0.036594972 |
| KEGG_ANTIGEN_PROCESSING_AND_PRESENTATION | -1.763545 | 0.032 | 0.040039435 |
| KEGG_PRIMARY_IMMUNODEFICIENCY | -1.7358152 | 0.029166667 | 0.047090225 |
| HALLMARK_DNA_REPAIR | 1.781226 | 0.014373717 | 0.039344862 |
| HALLMARK_G2M_CHECKPOINT | 2.1181214 | 0 | 0.003246718 |
| HALLMARK_MTORC1_SIGNALING | 2.1617544 | 0 | 0.001237374 |
| HALLMARK_MYC_TARGETS_V1 | 2.1529121 | 0 | 0.001209531 |
| HALLMARK_ALLOGRAFT_REJECTION | -1.8768002 | 0.017175572 | 0.11478418 |
| HALLMARK_IL2_STAT5_SIGNALING | -1.6324456 | 0.017821781 | 0.19343054 |
| GO_CYTOSKELETON_DEPENDENT_CYTOKINESIS | 2.1771717 | 0 | 0.023024553 |
| GO_MITOTIC_CYTOKINESIS | 2.1742215 | 0 | 0.015679935 |
| GO_PYRIMIDINE_NUCLEOSIDE_BIOSYNTHETIC_PROCESS | 2.155872 | 0 | 0.016314784 |
| GO_MITOTIC_NUCLEAR_DIVISION | 2.1444912 | 0 | 0.015454582 |
| GO_SISTER_CHROMATID_SEGREGATION | 2.1206279 | 0 | 0.014024315 |
| GO_DNA_DEPENDENT_DNA_REPLICATION | 2.0573618 | 0 | 0.028944924 |
| GO_CELL_CYCLE_G2_M_PHASE_TRANSITION | 2.0490954 | 0 | 0.030061029 |
| GO_ADAPTIVE_IMMUNE_RESPONSE | -2.1971807 | 0 | 0.038390122 |
| GO_B_CELL_RECEPTOR_SIGNALING_PATHWAY | -2.1852183 | 0 | 0.02490243 |
| GO_MAST_CELL_MEDIATED_IMMUNITY | -2.1464195 | 0 | 0.03642796 |
| GO_REGULATION_OF_LEUKOCYTE_MEDIATED_CYTOTOXICITY | -2.1418664 | 0 | 0.02878968 |
| GO_MAST_CELL_ACTIVATION | -2.1011052 | 0 | 0.04594304 |
| GO_LYMPHOCYTE_DIFFERENTIATION | -2.0913506 | 0 | 0.045823842 |
| GO_REGULATION_OF_LEUKOCYTE_MEDIATED_IMMUNITY | -2.0851812 | 0 | 0.03389096 |
| GO_T_CELL_DIFFERENTIATION | -2.0678575 | 0 | 0.032255348 |
| GO_REGULATION_OF_CELL_KILLING | -2.0604193 | 0 | 0.03441888 |
| GO_IMMUNE_RESPONSE_REGULATING_SIGNALING_PATHWAY | -2.0178487 | 0.001956947 | 0.02506339 |
| GO_T_CELL_ACTIVATION | -2.0103006 | 0 | 0.025686314 |
| GO_B_CELL_PROLIFERATION | -2.0076122 | 0.001988072 | 0.02584365 |
| GO_ANTIGEN_RECEPTOR_MEDIATED_SIGNALING_PATHWAY | -2.0073452 | 0 | 0.025236344 |

**
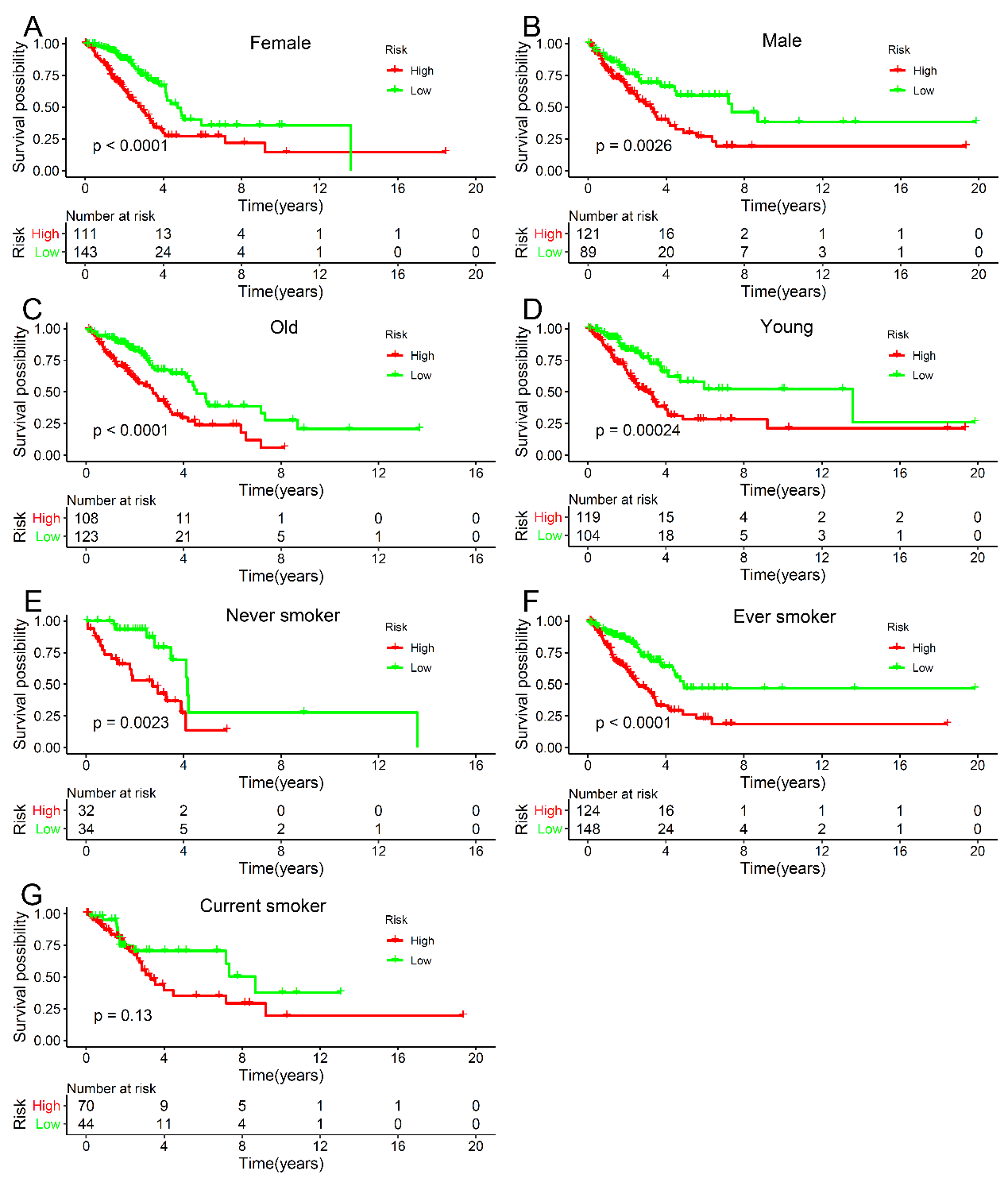
**

**Supplementary Figure S1**.Validation of predictive value of the five- IL(R)-based signature layered by clinical subtypes in TCGA cohort. Over survival curves for female **(A)**, male **(B)**, old (age>65) **(C)**, young (age≤65) **(D)**, never smoker **(E)**, ever smoker **(F)**, and current smoker **(G)** based on risk score in LUAD.

**
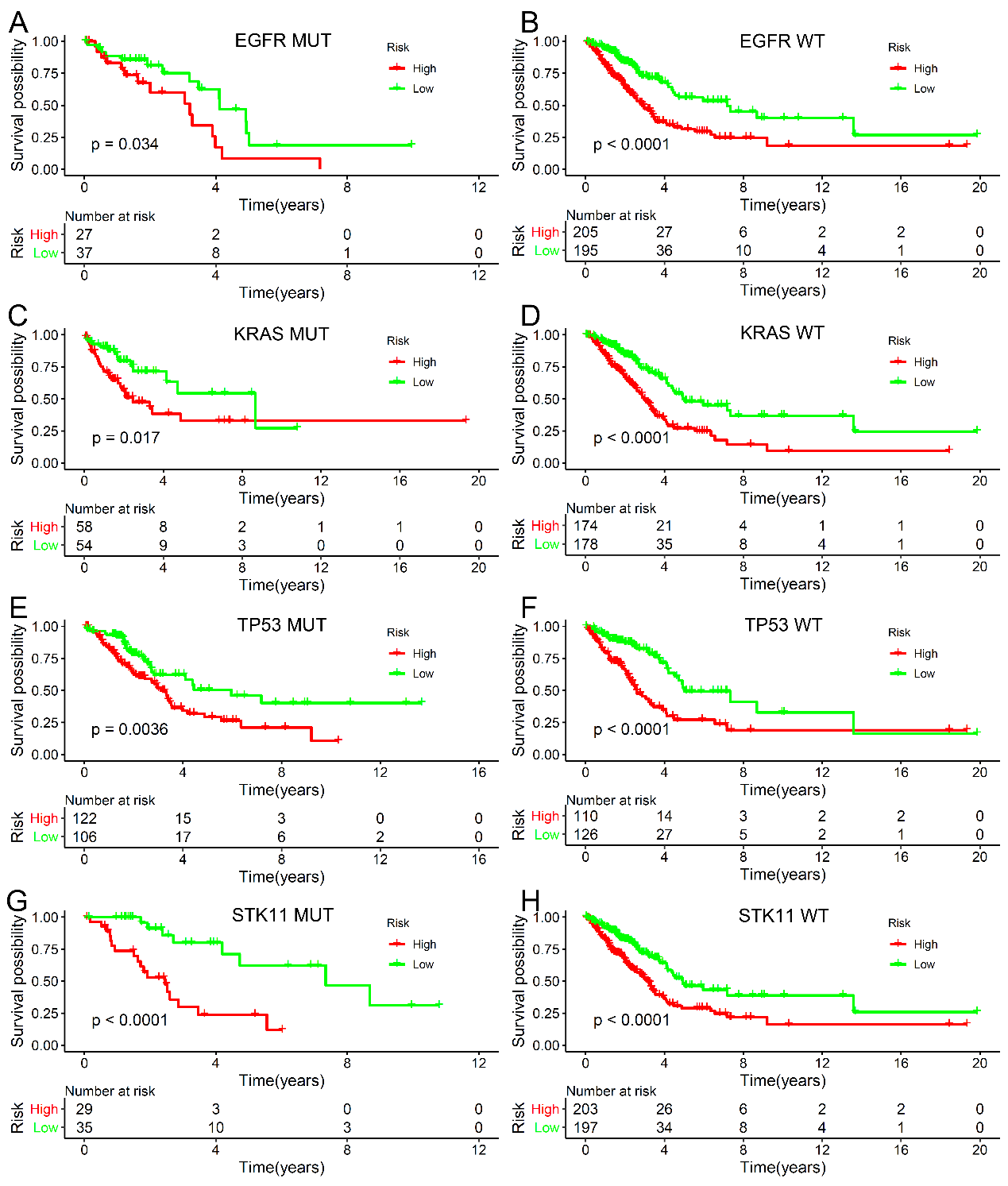
**

**Supplementary Figure S2**. Validation the prognostic performance of this IL(R)-based signature stratified by different gene mutation status. Over survival curves for patients with EGFR-MUT (**A**), EGFR-WT (**B**), KRAS-MUT (**C**), KRAS-WT (**D),** TP53-MUT (**E**), TP53-WT (**F**), STK11-MUT (**G**), and STK11-WT (**H)**based on risk score in LUAD population.


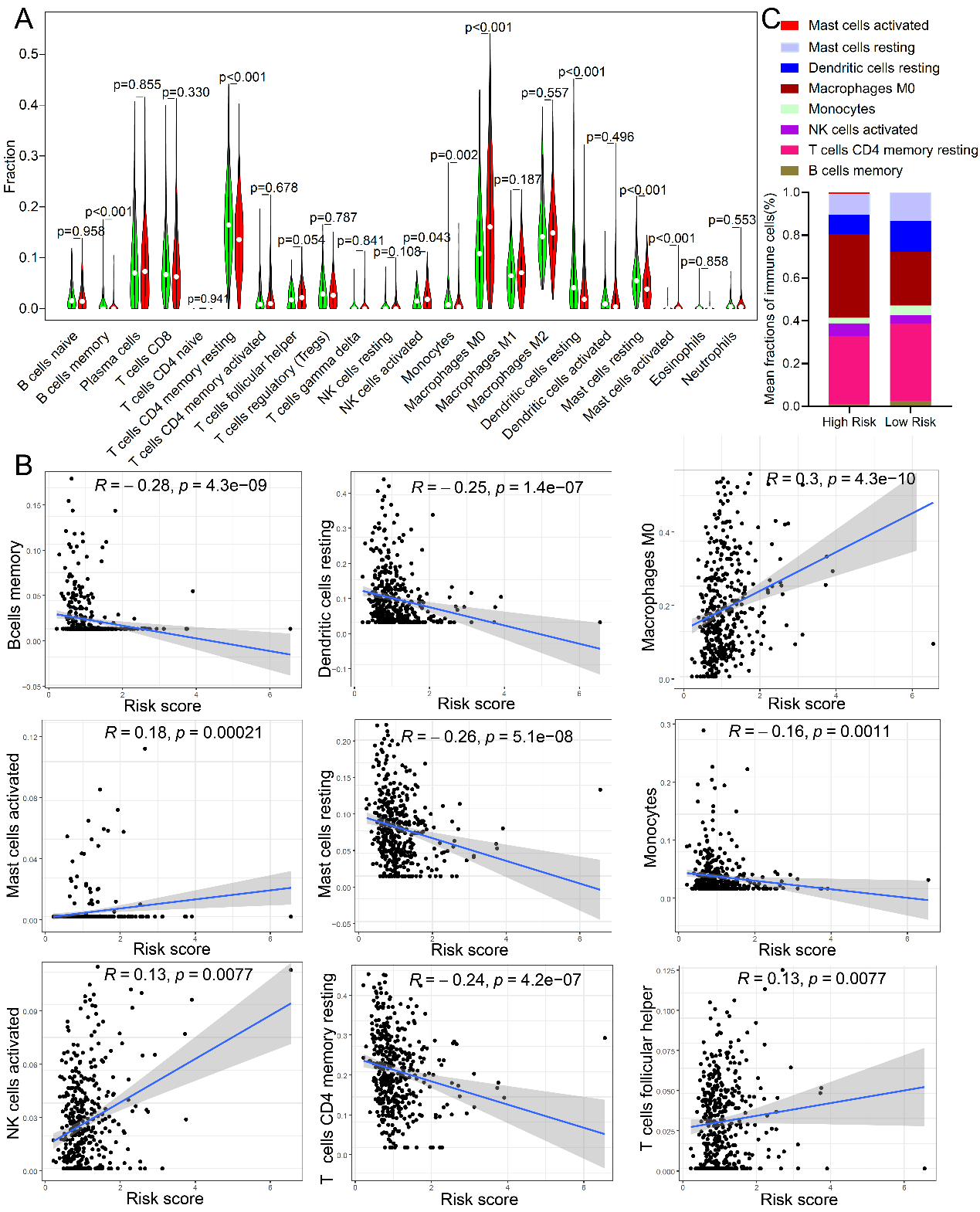


**Supplementary Figure S3.** The immune cell infiltration profile of the five-IL(R)-based signature in TCGA cohort. (**A**) Comparison of immune cell infiltration proportion in high- and low-risk groups. (**B**) Correlation analysis of between immune cells infiltration level and IL(R)-based risk score. (**C**) The distribution of immune cells obtained from intersection of correlation analysis and difference analysis

**
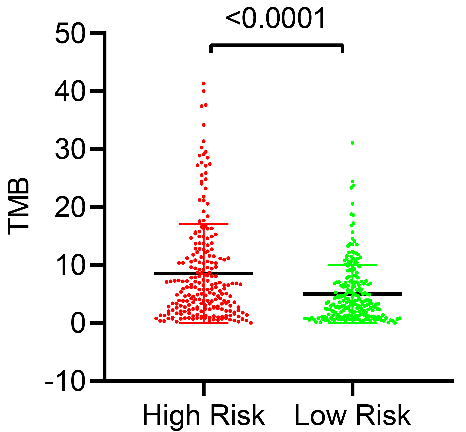
**

**Supplementary Figure S4**. The relationship between this IL(R)-based signature and TMB in LUAD population.

**
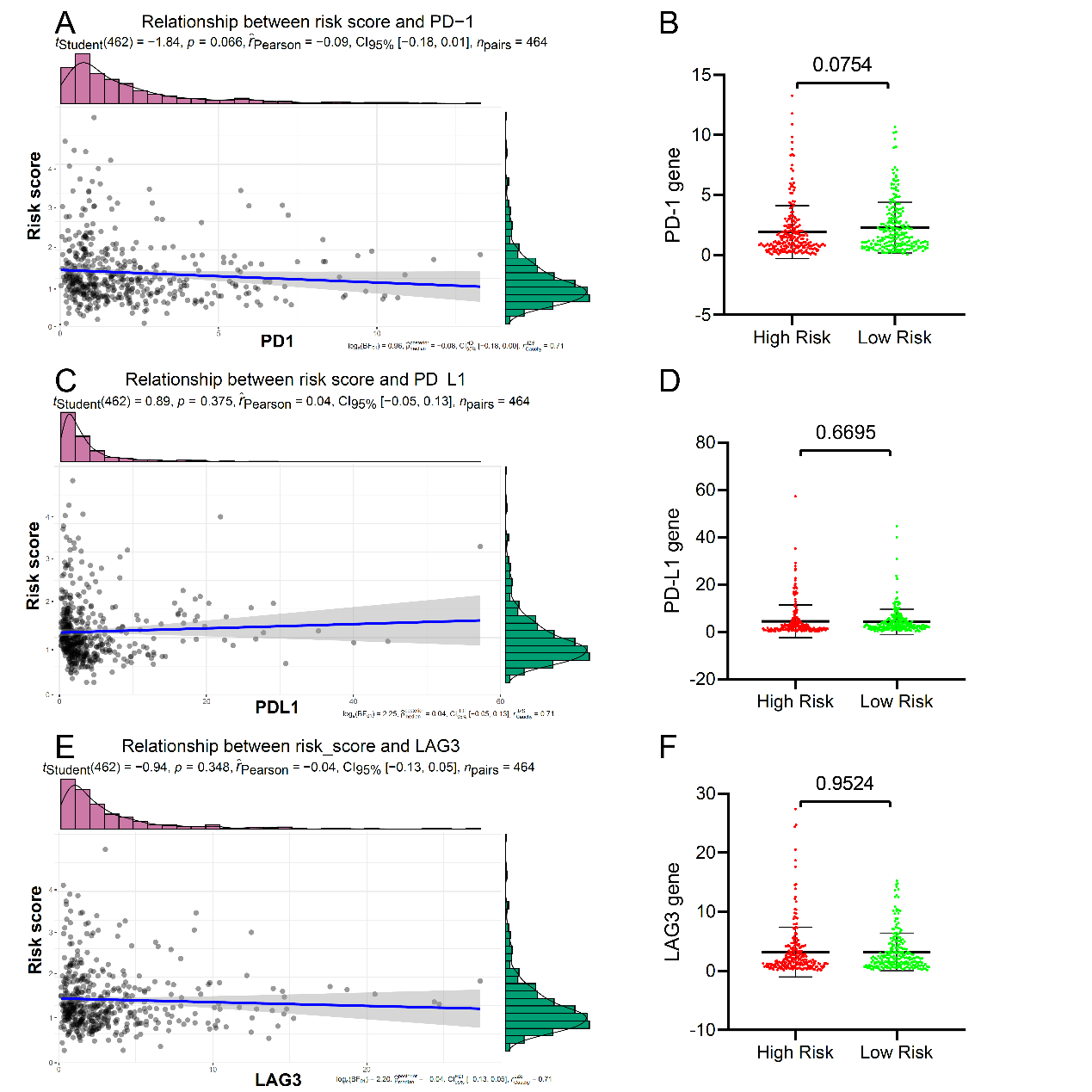
**

**Supplementary Figure S5**. Correlation and comparison analysis of IL(R)-based signature and immune checkpoints. Correlation of the IL(R)-based risk score and PD-1(**A**), PD-L1(**C**), and LAG3(**E**). Comparison of the IL(R)-based risk score and PD-1(**B**), PD-L1(**D**), and LAG3(**F**).

**
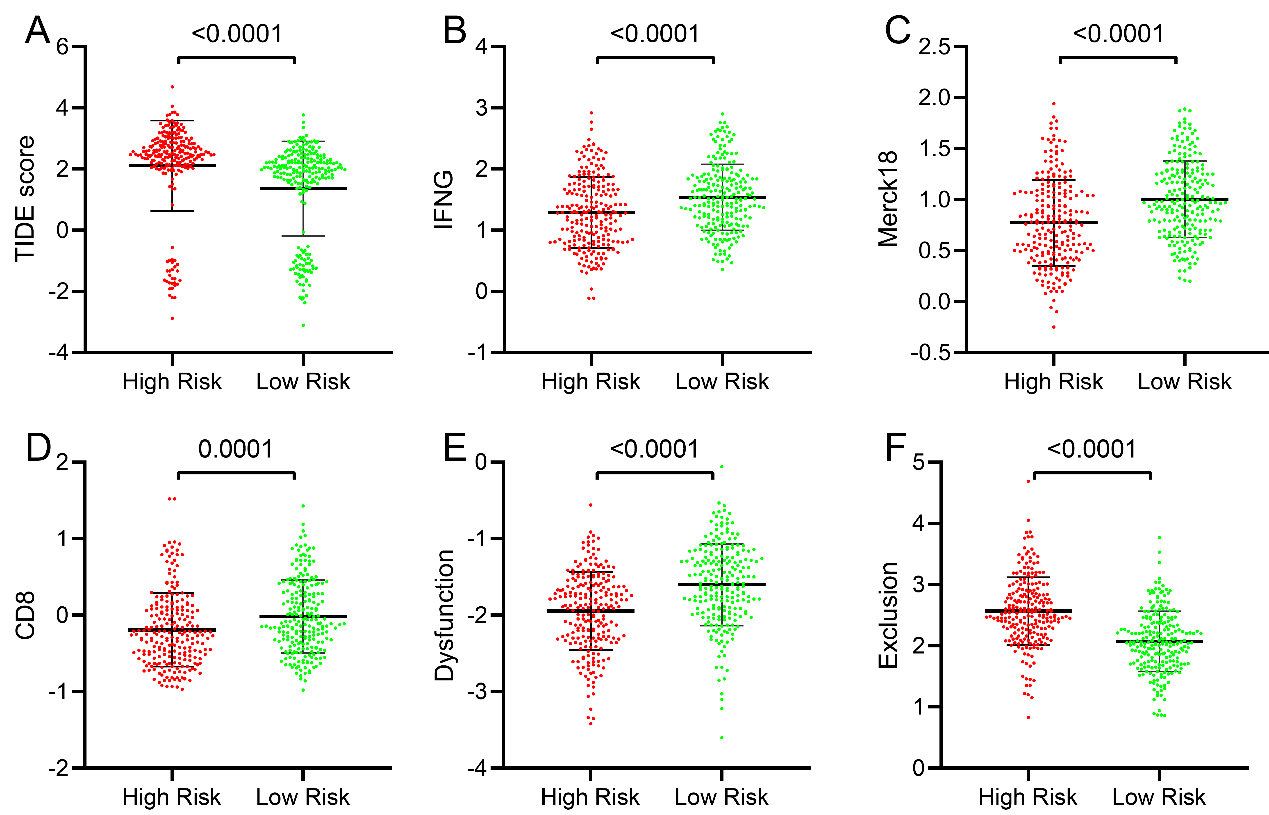
**

**Supplementary Figure S6**. Distribution of TIDE in the five- IL(R)-based signature. The distribution and comparison of TIDE score (**A**), IFNG (**B**), Merck 18 (**C**), CD8(**D**), T cell dysfunction score (**E**), and T cell exclusion score (**F**) in the high-risk group and the low-risk group.
